# Aggressive immunosuppressive treatment of Susac's syndrome in an adolescent: using treatment of dermatomyositis as a model

**DOI:** 10.1186/1546-0096-6-3

**Published:** 2008-01-29

**Authors:** Robert M Rennebohm, Martin Lubow, Jerome Rusin, Lisa Martin, Deborah M Grzybowski, John O Susac

**Affiliations:** 1 Department of Pediatrics, Division of Pediatric Rheumatology, Ohio State University College of Medicine, Columbus, Ohio, USA; 2 Department of Ophthalmology, Ohio State University College of Medicine, Columbus, Ohio, USA; 3 Department of Radiology, Nationwide Children's Hospital, Columbus, Ohio, USA; 4 Neurology and Neurosurgery, PA, Winter Haven, FL, USA

## Abstract

We describe aggressive immunosuppressive treatment of an adolescent with Susac's syndrome (SS), a disease of the microvasculature in the brain, retina, and inner ear. Because the immunopathogenesis of SS appears to have much in common with that of juvenile dermatomyositis (JDM), the patient was treated with an approach that has been effective for severe JDM. The patient's outcome provides evidence for the importance of prompt, aggressive, and sustained immunosuppressive treatment of encephalopathic SS.

## Introduction

Susac's syndrome (SS), which consists of the clinical triad of encephalopathy, branch retinal artery occlusion (BRAO), and hearing loss (HL), appears to be due to autoimmune endotheliopathy in the microvasculature of the brain, retina, and inner ear [1,2]. Resultant ischemic injury produces pathognomonic central corpus callosal "holes" (infarcts) on MRI [3], retinal infarcts that are associated with a distinctive multifocal staining on fluorescein retinal angiography [4], and HL that is often accompanied by roaring tinnitus and vertigo.

The immunopathogenesis of SS [1,2,5-9] appears to have much in common with that of juvenile dermatomyositis (JDM) [10-13], an autoimmune endotheliopathy that affects the microvasculature of a different triad of tissues (muscle, skin, and gastrointestinal tract). We report a 16 year old female with encephalopathic SS who was successfully treated with a protocol [2,14] based on aggressive treatment of severe dermatomyositis [13,15].

## Case presentation

Over a period of two weeks in January 2005, a 16 year old Caucasian female developed incapacitating headaches, recurrent vomiting, visual disturbance ("seeing round, dark black spots"), hearing loss, roaring tinnitus, slurred speech, unsteady gait, mild neck stiffness, confusion, short term memory deficit, slow thought processing, emotional lability, personality change, and a tendency to sleep excessively.

When seen 2 weeks into the course of her illness, her visual acuity was 20/20 OU, but dilated ophthalmologic examination revealed a subtle "cotton wool" spot in her right eye. Though tired and mildly lethargic, she conversed normally and was able to provide her own history. Her gait was unsteady. She had bilateral long tract findings and difficulty performing rapid alternating movements, particularly on the left. The rest of her physical exam was normal.

**Figure 1:**
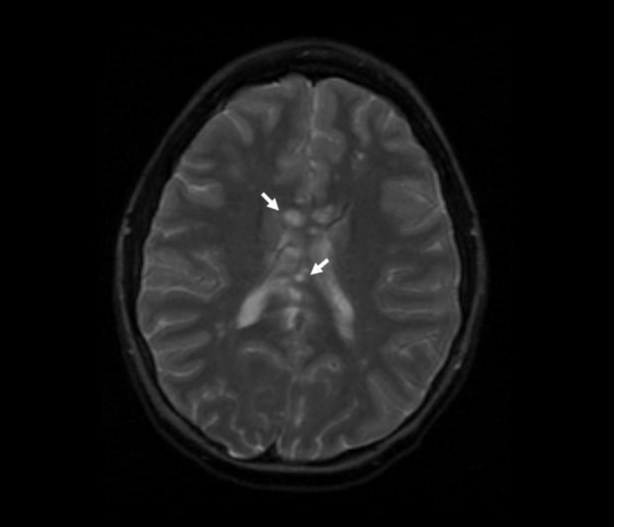
**MRI brain**. Axial T2 image showing multiple white matter lesions, especially in the corpus callosum. The arrows point to only two of several callosal lesions. Early in the disease, callosal lesions are usually best seen on thin section sagittal FLAIR and sagittal T1 images, with contrast.

MRI of the brain showed numerous widespread small hyperintense white matter lesions on T2 and FLAIR in both the supratentorial and infratentorial compartments. Several of these lesions enhanced, and there was impressive leptomeningeal enhancement cloaking the cerebral and cerebellar hemispheres. The central corpus callosum was extensively involved (Figure 1). Similar lesions were present in the basal ganglia. Diffusion weighted images showed restriction in many of these lesions.

Cerebrospinal fluid examination revealed an elevated protein (166 mg/dl), mild pleocytosis (11 WBC/dl, all lymphocytes), and an elevated myelin basic protein (28.54 ng/ml, with upper limit being 4.1 ng/ml). The CSF IgG index was normal. There were no oligoclonal bands. Her ESR, CRP, ANA, rheumatoid factor, muscle enzymes, and anti-phospholipid antibodies were normal or negative. Von Willebrand Factor-Antigen (VWF-Ag) and Factor VIII levels were not obtained prior to initiation of corticosteroid therapy, but were subsequently repeatedly elevated, as high as 530% and 330%, respectively (with normal being 50–150%). Early in her course she had mild thrombocytopenia (99,000–117,000).

**Figure 2:**
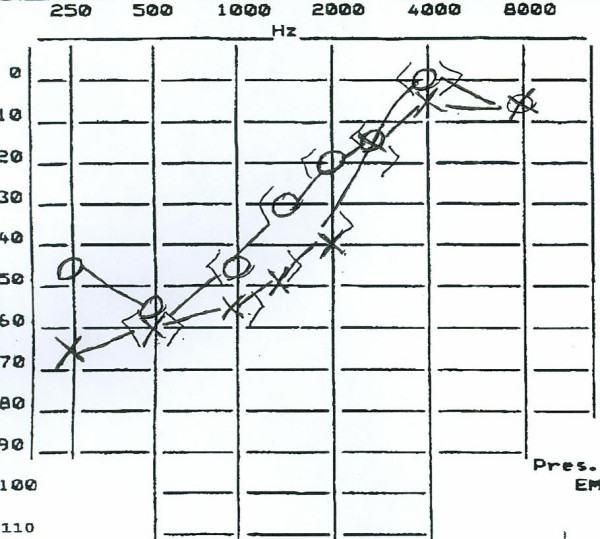
**Audiogram**. Audiogram showing bilateral low frequency hearing loss, rising to normal at higher frequencies. This is the typical (cochlear) hearing loss seen in SS. It likely reflects microinfarction of the striae vascularis at the apex of the cochlea. (X = left ear; O = right ear.)

Fluorescein retinal angiography showed a pattern compatible with resolved BRAO in the right eye and also revealed multifocal arteriolar disease in the retinal periphery. Audiograms (Figure 2) showed bilateral low frequency sensorineural hearing loss. Speech discrimination was impaired.

Formal neuropsychological evaluation revealed a Full Scale IQ of 104. Her estimated pre-morbid IQ was 121. She had poor verbal fluency, mild aphasia, short term memory deficits, slow processing speed, and executive dysfunction.

Although the MRI findings were initially thought to represent "atypical ADEM (acute disseminated encephalomyelitis)" or "atypical MS (multiple sclerosis)," the central location of the corpus callosal lesions, the involvement of the basal ganglia, and the presence of leptomeningeal enhancement led to a correct diagnosis of SS, especially when these MRI findings were coupled with the BRAO and HL.

**Figure 3:**
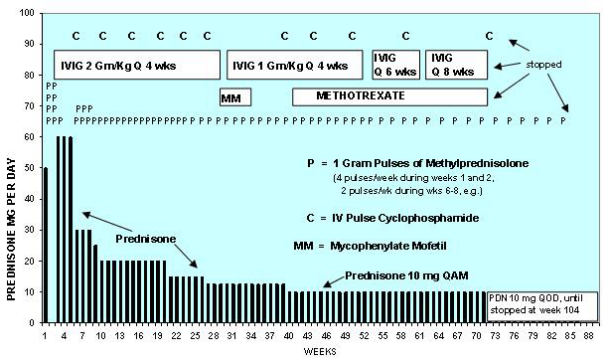
**Immunosuppressive therapy**. Graphic depiction of the patient's immunosuppressive treatment.

She was aggressively treated as shown in Figure 3. Initially, she received high dose oral prednisone, frequent pulses of methylprednisolone, monthly IVIG, and monthly pulses of IV cyclophosphamide. After receiving 6 monthly pulses of cyclophosphamide, she was placed on mycophenylate mofetil (MM) for maintenance therapy. However, since she was unable to tolerate side effects of MM (malaise and nausea), pulse cyclophosphamide therapy was resumed. Not shown in Figure 3 is a single dose of natalizumab that she received during the first week of treatment. Shortly after that single dose, we decided that the risk/benefit ratio for natalizumab did not justify further doses. Since then, we have not used natalizumab for this patient or any subsequent SS patients.

### Clinical Course and Outcome

**Figure 4:**
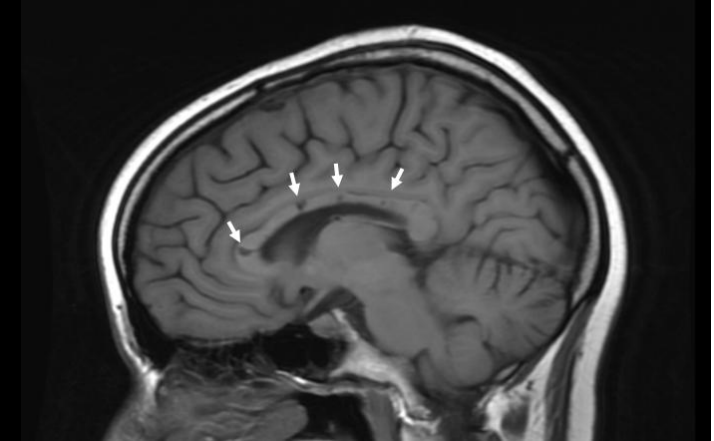
**MRI brain**. Sagittal T1 image showing the pathognomonic central callosal "holes" (microinfarcts) of SS. These residual "holes" (and sometimes, "spokes") develop as the acute callosal changes resolve.

The patient has now been followed for 36 months (156 weeks). Within two days after onset of treatment her headache and vomiting resolved, and her encephalopathy began to improve. Her encephalopathy continued to steadily improve thereafter and has never relapsed. Repeat neuropsychological evaluation at week 41 revealed a full scale IQ of 122 and a return to pre-morbid estimates in many domains. Fifty two weeks after onset of treatment she scored in the 98^th ^percentile on her college entrance examination. Repeat MRI at 60 weeks revealed marked resolution of the previously noted white matter lesions. Her corpus callosum was thinned and showed the typical residual callosal "holes" of SS (Figure 4).

Her BRAO quickly resolved without further visual complaints and without recurrence. Her vision at 41 weeks was 20/20 OU, with only a tiny asymptomatic field defect in the right eye. Her audiogram improved modestly during the first two weeks of treatment, but has not changed since that time. Her tinnitus was very distressing during the first 8 weeks, but has been quite tolerable since.

Her cyclophosphamide, IVIG and methotrexate were successfully tapered and discontinued by week 72. Pulses of methylprednisolone were gradually given less frequently and discontinued by week 84. She was tapered off of prednisone by the end of week 104 and has been on no medications since then.

At last follow-up (36 months) she still had residual, unchanged hearing loss and mild tinnitus, but her over-all neuropsychological status was better than that documented at week 41. She still had subtle short term memory difficulty and mild difficulty with executive function, but both of these problems had not changed since discontinuation of medication and appeared to represent residual deficits. No other residual deficits from her SS were evident.

Although we and the family did not think there was any ongoing active disease at the 3 year mark, the patient was struggling, emotionally, to cope with her permanent hearing loss and the above-mentioned residual cognitive deficits. These residual deficits have been heartbreaking and of great concern to the family. For this reason, we and the family feel her outcome has been good, but not excellent. Despite her residual deficits and the emotional challenges they have created, she is currently succeeding in a high honors curriculum in the humanities at a major university.

## Discussion

Our patient is the third reported pediatric patient who has experienced all three components of the SS triad. Hahn reported SS in a 16 year old female [16]; Muttikkal in a 9 year old girl [17]. Delaney reported an 8 year old child who had BRAO and hearing loss, but no clinically evident encephalopathy [18]. Of the approximately 80 cases of SS that have been reported in the international medical literature since Dr. Susac's first report in 1979, our patient represents only the second case of SS (adult or pediatric) reported in the rheumatologic literature [19]. Although elevated VWF-Ag levels have not been reported in SS, one report has mentioned elevated Factor VIII levels [8]. Thrombocytopenia has not been mentioned in any previous reports on SS.

Patients with SS often do not exhibit the complete triad at the beginning of their illness. Encephalopathy, BRAO, and hearing loss can each be the sole presenting manifestation of SS, with the other two components evolving later. Some patients with SS never develop clinical evidence of all 3 components of the triad.

Patients with SS are commonly mistakenly thought to have either "atypical multiple sclerosis (MS)" or "atypical ADEM." Several realizations can help the physician to distinguish SS from these two disorders [3]: In MS and ADEM the callosal involvement is along the undersurface of the callosum at the septal interface; while in SS the central fibers of the corpus callosum are primarily affected. Leptomeningeal enhancement, which occurs in 33% of patients with SS, does not occur in MS or ADEM. Deep gray matter involvement is rare in MS, though common in pediatric ADEM. Deafness is rare in MS and has not been reported in ADEM. True BRAO is incompatible with a diagnosis of MS, and has not been reported in ADEM.

SS should also be considered when a patient presents with an enigmatic encephalopathy (including encephalopathy with a predominance of psychiatric manifestations), or visual disturbance, or hearing loss (or combinations of these three) and has a working diagnosis of "possible neuropsychiatric lupus," "possible primary angiitis of the CNS," "unusual Meniere's disease," "autoimmune inner ear disease (AIED)," "possible Cogan's syndrome," "possible CNS (or retinal) vasospasm," "possible CNS (or retinal) embolism/thrombosis," or "unexplained branch retinal artery occlusion."

When SS is a consideration, it is essential to obtain an MRI of the brain and focus on the corpus callosum. Early in the disease, the typical callosal lesions are best seen on thin-section sagittal FLAIR and sagittal T1 (with contrast) images of the corpus callosum. Even if the patient has no obvious hearing loss or visual symptoms, it is important to obtain an audiogram and to consult a neuro-ophthalmologist/retinal specialist. Even if a careful fundoscopic exam is normal, we strongly urge fluorescein retinal angiography to look for asymptomatic retinal vasculopathy. When the angiography is performed, it is particularly important to include a careful look at the retinal periphery.

Brain biopsies, anatomical observations, and responses to immunosuppressive therapy suggest that SS is a primary autoimmune microvascular endotheliopathy: Most reports on brain biopsies of patients with SS have emphasized the finding of small foci of necrosis (microinfarcts) within the cerebral cortex and white matter. Unfortunately, only a few reports have provided detailed information about the microvasculature [5-9]. Fox reported a microangiopathy characterized by adventitial thickening and endothelial swelling in precapillary arterioles, thickening of the basal lamina, and a perivascular infiltrate in small vessels [5]. Heiskala reported that "the walls of several small arterioles were thickened and surrounded by an abnormal reticulin network and occasional lymphocytes; the normal capillary network was destroyed; and electron micrographs showed a remarkably thick basal lamina in many capillaries, both in the cerebral cortex and white matter [6]." Kaminska described "arteriolar wall proliferation with lymphocytic infiltration of the vessel wall [7]." Petty described "minimal perivascular lymphocytic infiltration without fibrinoid necrosis or necrotizing vasculitis [8]."

Petty performed muscle biopsies on 5 patients with SS and described "swollen endothelial cells and sparse periarteriolar inflammatory cells" in three [8]. "The swollen endothelial cells nearly occluded some small arterioles." Similarly, in a muscle biopsy of a patient with SS, O'Halloran described "occluded endomysial capillaries" and "foci of C5b-9 within the walls of small arterioles [9]." None of these patients had clinical evidence of muscle disease. Petty and others have suggested that the same microvascular endotheliopathy that is occurring (subclinically) in the muscle of patients with SS is also occurring in the brain, retina, and inner ear [8].

The above-described histopathologic and electron microscopic findings in the microvasculature of the brain and muscle of patients with SS are quite reminiscent of the microvascular abnormalities seen in the skin and muscle of children with juvenile dermatomyositis (JDM). For example, documented characteristics of the microvascular endotheliopathy of JDM include: endothelial cell swelling, sometimes to the point of lumenal occlusion; endothelial cell degeneration and necrosis; capillary network destruction ("drop-out"); basement membrane thickening, reduplication, and lamellation; granular C5b-9 deposition in blood vessels; and perivascular lymphocytic infiltration [10-12]. Interestingly, in JDM, microvascular endotheliopathy has also been noted (rarely) in the brain and retina [20,21]. For example, in JDM, the same endothelial cell swelling and endothelial cell necrosis that is seen in the muscle, skin, and GI tract has been noted (rarely) in the microvasculature of the brain [20]. Spectacular multifocal branch retinal artery staining on fluorescein angiography has also been seen in JDM [21]. And, transient retinal exudates and "cotton wool" spots, with temporary loss of vision, have been described in JDM [22].

In SS, the findings on fluorescein retinal angiography provide strong support for the notion that endothelial injury plays a central role in the pathogenesis of SS. The multifocal fluorescein staining/leakage localizes the site of immune attack to the endothelium of the precapillary arteriole (less than 100 microns), a site that consists primarily of endothelium and basement membrane. The elevated VWF-Ag and Factor VIII levels seen in our patient also provide evidence for endothelial cell perturbation in SS.

SS and JDM, therefore, have several characteristics in common. Both represent microvascular endotheliopathies that cause ischemic insult to a triad of tissues – primarily skin, muscle, and gastrointestinal tract in dermatomyositis; primarily brain, retina, and cochlea in SS. Both have biopsy findings characterized by endothelial cell injury in microvasculatures. Elevated VWF-Ag and Factor VIII levels have been detected in both diseases. Both follow variable courses [1,2,13,23,24]. In both cases, the disease may follow a 6–24 month duration monocyclic course (encephalopathic variety in SS), a polycyclic course (the recurrent BRAO/HL subset in SS), or a prolonged chronic continuous course (rare in SS). Both respond to immunosuppressive medication [1,2,13-15,23,24]. Finally, in SS, microvascular endotheliopathy can be found in muscle [5]; and, in JDM, microvascular endotheliopathy can be found in brain and retina [20,21].

Because SS and JDM appear to have much in common, we suggest that lessons learned from study of the immunopathogenesis, clinical course and treatment of JDM may be applicable to SS. With this in mind, we treated our patient with an aggressive immunosuppressive approach [2,14] that has been effective for severe JDM [13,15].

Although we believe her treatment served her very well, the varied and only somewhat predictable natural history of SS makes it difficult to know exactly how much credit her treatment should be given for suppressing and maintaining control over her disease. To date, our knowledge of the natural history of SS and its responsiveness to treatment is based entirely on anecdotal case reports and retrospective analysis of short series of patients [1,2,5-9,14,16,17,23]. No controlled studies of treatment have been conducted.

Analysis of reported cases reveals that some patients with SS, despite receiving no immunosuppressive treatment, have experienced a monocyclic course that has remitted spontaneously and resulted in good clinical outcome. (Such cases initially suggested that SS might not be an autoimmune disorder.) Other patients have seemed to respond dramatically to corticosteroid therapy – strongly suggesting that SS is an autoimmune disorder. Others have failed to respond adequately to corticosteroid therapy, but have seemed to respond well to cyclophosphamide – suggesting that SS sometimes needs more than just corticosteroid therapy. Some patients have failed to respond to both corticosteroid and cyclophosphamide therapy – suggesting that some patients may have such severe disease that usual corticosteroid and cyclophosphamide therapy may be inadequate. Many patients have responded well to immunosuppressive therapy, but have relapsed (either spontaneously or when immunosuppressive therapy has been tapered too quickly), and have again responded to reinstitution or escalation of immunosuppression.

The above case reports are reminiscent of what is known about the natural history of JDM and the spectrum of responsiveness of JDM to various immunosuppressive therapies. It is wise to recall that in the pre-steroid era, a third of JDM patients recovered fully without any treatment, a third experienced chronic disability, and a third died. It is also important to realize that the prompt, aggressive, and sustained immunosuppressive treatment that children with JDM now receive has resulted in a marked improvement in outcome [15]. At the current time, there is still a spectrum regarding how much immunosuppressive medication JDM requires (and for how long). The same is probably true for SS. Clearly, careful collaborative study of as large a number of patients as possible will be necessary to determine optimal treatment of SS.

In the meantime, we encourage prompt, aggressive, and sustained immunosuppressive treatment of SS. We think such treatment may prevent or minimize the feared sequelae of dementia, vision loss, and hearing loss. We suspect that, historically, encephalopathic SS has often been treated too late, with too little, and/or for too short a time. Historically, this was the case with JDM, too. We strongly encourage international collaborative study of SS to further test the hypothesis that the immunopathogenesis of SS is similar to that of JDM, and to determine optimal treatment. Our goal should be excellent outcome, not just good outcome. Until more is known, we offer the protocol depicted in Figure 3 and detailed elsewhere [2,14] as a tentative model for treatment of encephalopathic SS. Finally, since SS appears to be an immune-mediated disease with immunohistopathologic characteristics similar to those seen in JDM and requires long-term immunosuppressive therapy, we urge the international rheumatology community to become more aware of SS, to view it as an autoimmune disease, and to become more involved in its diagnosis, treatment, and study.

## Conclusion

Susac's syndrome, which consists of the clinical triad of encephalopathy, branch retinal artery occlusion, and hearing loss, appears to represent an autoimmune endotheliopathy in the microvasculature of the brain, retina, and inner ear (sometimes with subclinical involvement of muscle). The immunopathogenesis may have much in common with that of juvenile dermatomyositis, an autoimmune endotheliopathy that causes ischemic injury in a different triad of tissues – muscle, skin, and gastrointestinal tract (and, rarely, brain and retina). Because of these similarities, we believe that lessons learned from study of the immunopathogenesis, clinical course, and treatment of JDM may be applicable to SS. Accordingly, we treated our patient with an aggressive immunosuppressive approach that has been effective for severe JDM. She appeared to benefit considerably from this treatment. We strongly encourage international collaborative study of SS to further test the hypothesis that the immunopathogenesis of SS is similar to that of JDM and to determine optimal treatment. In the meantime, we encourage prompt, aggressive, and sustained treatment of SS, and we offer the protocol depicted in Figure 3 as a tentative model for treatment of encephalopathic SS.

## Competing interests

The author(s) declare that they have no competing interests.

## Authors' contributions

RR organized and wrote the majority of the manuscript and provided the bulk of the patient's treatment and care. ML and DG provided ophthalmologic interpretation, helped substantially with editing, and played a key role in the recognition and investigation of the notion that Susac's syndrome is an autoimmune endotheliopathy and has features in common with juvenile dermatomyositis. J R and LM provided interpretation of the patient's MRI, selected images for inclusion in the manuscript, wrote the captions for the MRI images, and provided radiological advice and editing for the manuscript. J S provided advice regarding the diagnosis and care of the patient, helped plan and organize the manuscript, and helped write and edit the manuscript. All authors participated in writing the manuscript and have read and approved the final manuscript.

## Consent

Written patient consent has been obtained for publication of the report.
